# Contrast bolus timing in CT-angiography and CT-perfusion: insights from a large clinical dataset

**DOI:** 10.1007/s00234-025-03558-5

**Published:** 2025-04-11

**Authors:** Alexander Rau, Samer Elsheikh, Petra Cimflova, Thomas Stein, Christian A. Taschner, Jonas A. Hosp, Marco Reisert, Horst Urbach, Elias Kellner

**Affiliations:** 1 Department of Neuroradiology, Medical Center– University of Freiburg, Faculty of Medicine, University of Freiburg, 79106 Freiburg, Germany; 2 Department of Diagnostic and Interventional Radiology, Medical Center– University of Freiburg, Faculty of Medicine, University of Freiburg, 79106 Freiburg, Germany; 3 Department of Neurology and Clinical Neuroscience, Medical Center– University of Freiburg, Faculty of Medicine, University of Freiburg, 79106 Freiburg, Germany; 4 Medical Physics, Medical Center– University of Freiburg, Faculty of Medicine, University of Freiburg, 79106 Freiburg, Germany; 5 Department of Stereotactic and Functional Neurosurgery, Medical Center– University of Freiburg, Faculty of Medicine, University of Freiburg, 79106 Freiburg, Germany; 6 Department of Neuroradiology, Freiburg University Medical Center, Breisacher Straße 64, 79106 Freiburg i.Br., Germany

**Keywords:** CT perfusion, Stroke imaging, Bolus timing, CT angiography

## Abstract

**Introduction:**

CT-Perfusion (CTP) is an essential part of stroke imaging. Incomplete coverage of the contrast bolus in CTP can lead to errors in post-processing that might hamper the identification of the infarct core or tissue at risk. However, the arrival of the contrast bolus depends on various technical and patient individual factors. This study investigated whether timing information from CT-angiography (CTA) can be used to optimize bolus coverage in CTP.

**Methods:**

We retrospectively reviewed cases with a multimodal stroke protocol for suspected ischemic stroke. Information on the contrast injection timing of CTA and CTP was extracted from the DICOM headers. Bolus arrival information were obtained from the CTP scan including peak time, height, and width and correlated with patient age and ejection fraction (the latter available in *n* = 868). The contrast timing information of the CTA was used to simulate optimized CTP timing.

**Results:**

A total of 1,843 cases were included. CTP bolus peak position was associated with peak width (Pearsons’s *r* = 0.89, *p* < 0.001), age (Pearsons’s *r* = 0.40, *p* < 0.001), ejection fraction (Pearsons’s *r*=-0.25, *p* < 0.001), and time to scan initiation based on triggering in CTA (Pearsons’s *r* = 0.83, *p* < 0.001). Using information of the CTA timing to adjust the CTP timing, the variance of the AIF peak could significantly be reduced (*p* < 0.001).

**Conclusion:**

Our data indicate that patient individual characteristics lead to substantial variances in the contrast bolus arrival which could hamper CTP analysis. To ensure optimized coverage of the contrast bolus. CTP timing can significantly and safely be improved using timing information of preceding CTA.

## Introduction

Ischemic stroke constitutes a major cause of death and disability [1]. In acute stroke, imaging is crucial to differentiate between ischemic and hemorrhagic stroke and to guide treatment [1, 2]. Multimodal CT stroke imaging employs non-contrast CT for the evaluation of brain tissue, CT angiography CT (CTA) for the detection of vessel occlusion, and CT perfusion (CTP) to evaluate the viability of brain tissue including the assessment of the infarct core and tissue at risk [1, 2]. Both CTA and CTP imaging rely on the administration of contrast agents to visualize vascular structures and assess tissue perfusion. After contrast injection, the cranium is scanned at the timepoint of contrast agent bolus arrival either in a single scan for CTA or repeatedly in CTP. Complete temporal coverage of the bolus in CTP is essential for correct processing and to obtain reliable outputs regarding the infarct core and penumbra, i.e. the tissue at risk [3].

After the peripheral injection, the contrast bolus disperses within the heart and lung system and mixes with venous blood before reaching the scan region. This arrival of the contrast bolus in the neurocranium is subject to a specific delay and dispersion, influenced by various factors such as injection site, flow rate, and individual patient variables like cardiac output [4–7]. The optimal timing between contrast administration and scan initiation is crucial for achieving appropriate image contrast and diagnostic value [6, 8, 9]. CTA aims for high arterial contrast, so the singular acquisition should ideally occur shortly before or at the arterial peak, i.e. before venous filling. In CTP, the scanning needs to capture the entire passage of the contrast bolus through the brain tissue. Poor timing can result in insufficient contrast-to-noise ratios and contamination with venous signals in CTA, and bolus truncations in CTP [10, 11]. Moreover, insufficient bolus coverage hampers processing and can thus negatively influence treatment decisions. However, longer than necessary scan times substantially increase the radiation exposure [10, 12].

Various strategies were developed to ensure precise, patient-specific timing in imaging procedures. One such method is the “test-bolus” approach [7, 13, 14] which involves repeated low-dose and low-contrast scanning in a vascular region such as the aortic arch to generate a concentration-time curve. From these curves, the optimal time point for the CTA acquisition is determined. However, this technique requires an additional injection of contrast agent, an extra scan, and manual intervention by the operator. In contrast, the bolus tracking method offers a more streamlined alternative [7, 14]. In this approach, the imaging device monitors density values within a prespecified region of interest and automatically triggers the CTA scan once a predetermined threshold, typically set at 100 Hounsfield units (HU), is exceeded. Although not without limitations, such as potential inaccuracies in threshold determination, these automated methods provide a tailored, patient-specific approach to scan initiation, ensuring optimal timing to capture the arterial phase with the highest contrast.

**Fig. 1:**
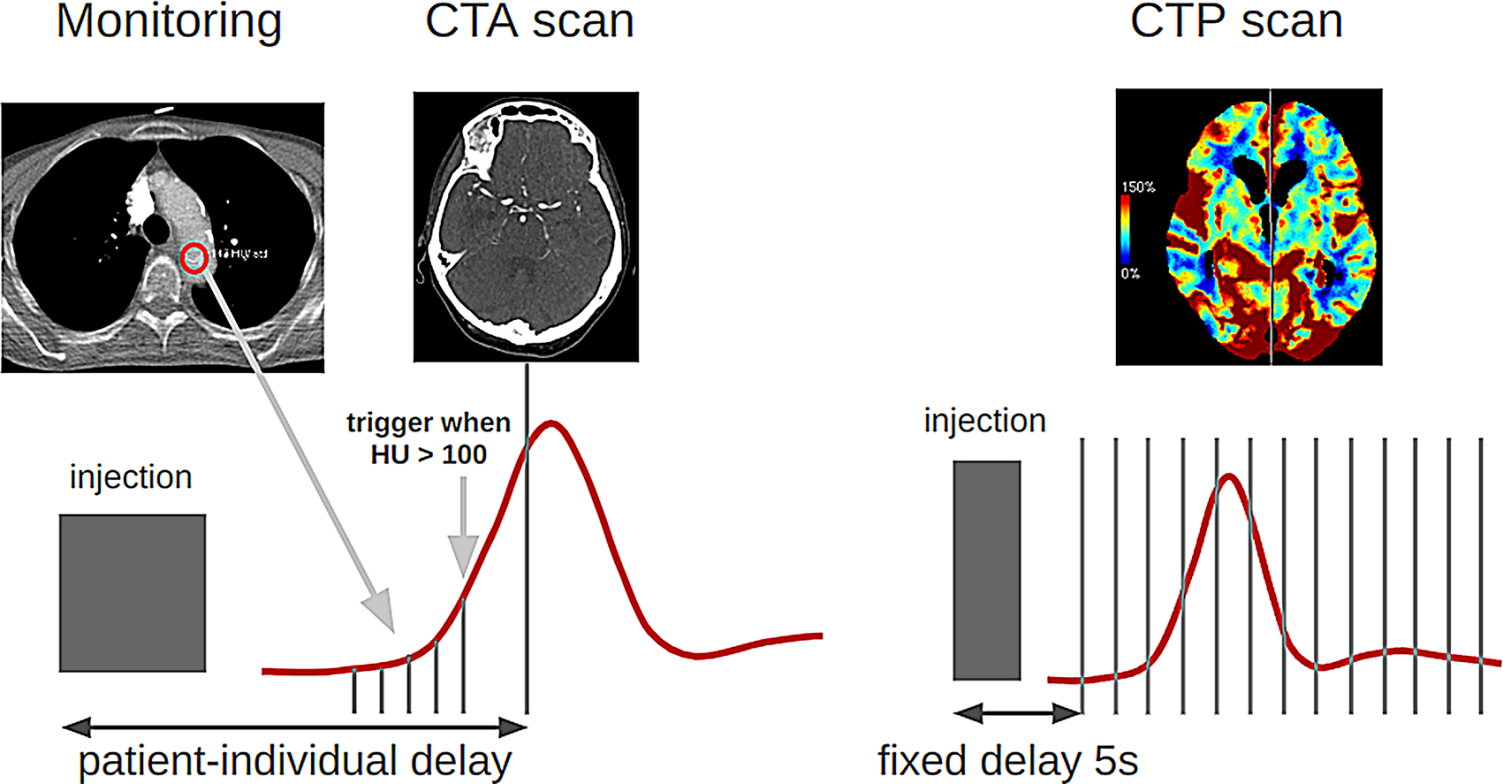
Workflow of a multimodal stroke imaging protocol. First, a region of interest (ROI) was manually placed in the aortic arch. After administration of the contrast agent, a repeated scan was acquired and the Hounsfield unit (HU) values in the ROI were automatically monitored. When the value exceeded the threshold of 100 HU, the CTA scan was initiated. In CTP, baseline images prior to contrast arrival were required for the calculation of perfusion metrics. This requirement conflicts with simultaneous bolus tracking in the aortic arch. Consequently, the CTP scan was consistently performed with a fixed delay of 5 s, serving as a compromise between early and late contrast arrivals. The red line depicts the bolus attenuation curve in the brain tissue

CTP relies on a series of sequential brain scans rather than a single scan at a specific phase, allowing for comprehensive perfusion data acquisition. This technique requires baseline scans to be performed before the arrival of the contrast bolus to establish a reference. However, it is not feasible to simultaneously acquire baseline brain images, track the bolus in the aortic arch, and capture the complete bolus passage in the brain. Consequently, CTP cannot be automatically triggered by bolus tracking. Instead, CTP scans are typically initiated after a fixed delay, usually around 5–10 s after the administration of contrast agent injection [15], see Fig. 1.

However, this fixed delay may not suit all scenarios. Cases with exceptionally early or late contrast arrival can experience truncated concentration curves, potentially leading to inaccuracies in perfusion assessments.

One promising solution involves leveraging patient-specific timing information obtained from the preceding CTA scan to inform CTP timing as proposed by Kasasbeh and colleagues [12]. The idea is to assess the patient-individual bolus characteristics from the CTA scan, and to adapt the CTP timing accordingly. For example, if the CTA bolus arrives late, the CTP delay should also be increased. Recent evaluations of such “rule-based” approaches have successfully employed refined CTP acquisition in patients with low cardiac output [10] and retrospectively assessed reduced CTP scan durations in patients with rapid bolus arrival in CTA [16].

Although technically feasible, this strategy did not yet reach clinical routine. In our study, we sought to bridge this gap by analyzing a large clinical dataset to explore the potential benefits of incorporating CTA-derived bolus information into CTP timing. We hypothesized that such an optimized acquisition timing could improve bolus coverage in CTP while also providing insights into CTA bolus dynamics.

## Materials and methods

### Participants

We included all consecutive patients presenting with symptoms of acute ischemic stroke who underwent multimodal imaging, including CTA and CTP, between 2014 and 2021 in this retrospective study. As part of the data management process, all imaging data were anonymized and exported to a local instance of the imaging platform NORA (www.nora-imaging.org). Patients with non-ischemic lesions such as tumors or hemorrhages were excluded. Quality assessment was performed individually for all imaging data and cases with significant image artifacts, such as severe movement artifacts or insufficient/failed contrast administration, were excluded from further analysis.

Cardiac function was assessed via the ejection fraction in cases where echocardiography was available in clinical documentation.

The study was approved by the Institutional Review Board (Ethics Committee– University of Freiburg; EK 20/1047) and carried out in accordance with the Declaration of Helsinki and its later amendments. The need for written informed consent was waived due to the retrospective nature of this study.

### CT imaging

CT scans were performed on a 128-detector row CT scanner (Somatom Definition Flash, Siemens Healthineers, Forchheim, Germany).

CTA scans were obtained in spiral mode covering the aortic arch to the vertex with the following settings: reference tube voltage = 100 kV, reference tube current = 155 mAs, CARE Dose4D, collimation = 128 × 0.6 mm, tube rotation time = 0.28 s, no gantry tilt. CTP series were acquired in spiral mode with the following settings: 80 kV, 180 mAs, collimation = 16 × 1.2 mm, tube rotation time = 0.3s, no gantry tilt, slice thickness = 5 mm, z-coverage = 100 mm, 27 series every 1.5s = 42s total scan time.

#### Contrast injection

Contrast agent was administered with an Accutron^®^ CT-D Vision (Medtron^®^, Saarbrücken, Germany) into a cubital line (16–18 G) with the following settings: CTA: contrast agent dose = 70 mL, flow rate = 5 mL/s, duration = 14s, followed by a 60 mL saline flush at a flow rate of 5 mL/s. CTP: contrast agent dose = 35–40 mL, flow rate = 6 mL/s, duration = 6.6s, followed by a 30 mL saline flush at a flow rate of 5 mL/s. The reported flow rates and durations represent nominal values. Actual values may vary if the injector pressure limit (325 psi) is exceeded.

#### Scan timing

For CTA acquisition, the bolus tracking method was employed; following the administration of the contrast agent, a scan in the aortic arch was repeated every second, and the HU value was automatically monitored in a manually preselected region of interest in the ascending aorta. Once the value exceeded a threshold of 100 HU, the CTA scan was triggered (due to table movement and scanner recalibration requirements, the actual CTA scan acquisition was initiated 4 s after this point). This approach ensured a customized delay between contrast injection and the commencement of the CTA scan tailored to each patient’s physiology. In contrast, CTP scans were consistently initiated with a fixed delay of 5 s.

### Extraction of DICOM information

We extracted the following tags from the DICOM headers of all CTA and CTP scans: “Contrast Bolus Volume”, “Contrast Bolus Stop Time”, “Contrast Bolus Total Dose”, “Contrast Flow Rate”, “Contrast Flow Duration” and “Acquisition Time” of the first and last image. The “Contrast Bolus”- related tags were not always present in the DICOM headers, presumably due to incompatibility in data transfer between the injector and CT scanner [17]. Cases with missing “Contrast Bolus” tags were excluded from further analysis. Furthermore, cases with injection schemes differing from the protocols described above were excluded. In the next step, the patients’ individual “Scan Delay” of the CTA was calculated as the time between the “Contrast Bolus Start Time” and “Acquisition Time” of the first image. The CTA trigger time, i.e. the time point when the threshold of 100 HU in the aortic arch was first exceeded, was calculated as the “Scan Delay” minus 4 s since the CTA was initiated 4 s after that time (see scan protocol above). The “Scan Duration”, i.e. the actual data acquisition window, was calculated as the time from the first to the last image.

### Analyzed parameters of the CTA and CTP acquisition/timing

#### CTP bolus characteristics

The CTP arterial input function (AIF) was automatically derived using the perfusion software VEOcore (VEObrain GmbH, Germany). A gamma variate function was fitted to the curve to remove noise and obtain stable parameters. From the fitted curves, the AIF peak position and peak width (full width at half maximum, FWHM) were obtained. To ensure that the CTP scans from the clinical routine had sufficient quality (i.e. not impaired due to very low contrast bolus, no artifacts from severe patient movement, no bolus truncations, no contrast bolus interference with the preceding CTA scan [18], and others), visual and automated quality control of the perfusion results and fitted AIF curves was performed based on the following criteria: Chi^2^ from fit < 0.1, FWMH < 24s, peak position relative to the acquisition window < 0.9, peak height < 5HU.

#### Optimized CTP timing

From a technical point, the CTP AIF peak should ideally occur shortly before the center of the acquisition window. This is not always achievable utilizing a fixed scan delay due to patient-individual variability in bolus arrival [19]. We hypothesized that the timing of CTP acquisition can be improved by incorporating the trigger time from the preceding CTA scan as a patient-individual delay for the CTP scan. In clinical practice, such an approach could be based on software implementations by vendors to automatically forward the CTA trigger time into the CTP scan protocol. Alternatively, a manual “rule-based” approach could be employed. For the latter, the technician manually chooses between several predefined CTP delays, depending on the information from the CTA scan. We investigated the feasibility of both approaches: For the automated approach, we used the following formula: *D*_*CTP*_*= T*_*CTA*_*− 15s*, in which D_CTP_ denotes the CTP delay and T_CTA_ denotes the trigger time from the CTA scan. The 15s were empirically chosen to ensure that, on average, the CTP delay remains within a similar timeframe as the fixed 5-second delay, thus allowing for an adequate capture of a sufficiently long baseline. For the rule-based approach, we tested the performance of the following rules: (a) Default: CTP delay 5s, (b) CTA trigger < 14s: CTP delay of 0 s, or (c) CTA trigger > 24s: CTP delay of 12 s.

#### Optimized CTA timing

Optimal arterial contrast enhancement of arteries in CTA requires an acquisition shortly before or at the arterial peak, which is not always the case in clinical practice [20]. The optimized CTA timing was tested by several approaches. First, we simulated the theoretical CTA arterial attenuation curve by comparing the CTA scan time with the AIF derived from CTP. While doing so, the different injection schemes (14s duration at 5 mL/s for CTA and 6.6s duration at 6 mL/s for CTP) were also taken into account.

We simulated the theoretical CTA arterial attenuation curve by convolving the CTP AIF with a box function with a width of 7.4s, corresponding to the difference between CTA and CTP injection durations. Of note, the chosen width of 7.4s was only an approximation; since the injection schemes are not fully rectangular in practice [21], we consider this sufficient for the current analysis. The difference between the actual CTA scan time and the peak of the simulated CTA arterial attenuation curve was calculated (Δ_CTA_).

We hypothesized that CTA scans were triggered either too early, accurately, or too late. To confirm this assumption, we chose 30 scans based on Δ_CTA_ (10 scans with greatest negative Δ_CTA_, 10 scans with Δ_CTA_ = 0, and 10 scans with greatest positive Δ_CTA_) for the visual assessment of the optimal CTA timing. Two experienced interventional neuroradiologists independently assessed arterial and venous contrast enhancement in these 30 scans. Intravascular enhancement in the right distal ICA and M1 and in the right transverse and sigmoid sinus were rated separately on 5-point Likert scales (0 = no enhancement, 1 = contrast agent barely conceivable, 2 = weak enhancement, 3 = good enhancement, 4 = perfect enhancement). Endoluminal Hounsfield units (HU) were measured in the right distal ICA and right transverse sinus in the aforementioned 30 patients.

### Statistical analysis

All statistical analyses were performed using MATLAB (the Mathworks) and R (Version 4.2.1; https://www.R-project.org*).* Baseline clinical characteristics were summarized using descriptive statistics.

#### CTP bolus characteristics

CTP AIF peak positions and peak widths were correlated to determine the association between bolus delay and dispersion. A multivariate linear regression model was adjusted for age, sex, weight, and ejection fraction to elucidate the individual contributions of these variables to the CTP AIF bolus peak position.

#### Optimized CTP timing

The impact of the adjusted CTP timing was assessed by analyzing the variance of the distributions of the peak positions with and without correction, and by outlier count.

Outliers were defined as either those with too early or too late timing when the AIF peak occurred 15s or later than 30s after the injection start. An AIF peak position occurring less than 10 s after the scan start (equivalent to 15 s after injection start in our protocol) was deemed too early due to the insufficient duration of a baseline required for CTP calculation. Conversely, a peak occurring 25 s after the scan initiation (equivalent to 30 s after injection start) was considered too late, as there was inadequate scanning time left to capture delayed curves from hypoperfused areas in case of vessel occlusion.

#### Optimized CTA timing

To visualize the optimal CTA timing, the distribution of Δ_CTA_ was plotted together with the mean of all CTA attenuation curves centered around the max position. Differences in visually evaluated scores and measured attenuation (HU) were compared using the Mann-Whitney U based on the average scores of the two readers per reading item for accurate vs. early or late bolus peak position. Additionally, ANOVA was used to compare the arterial and venous endoluminal attenuation across the groups with Tukey’s post hoc test.

## Results

### Participants

We identified 2203 eligible patients with CTA and CTP imaging. Of those, 1894 followed the standard injection protocol described above and 51 did not pass the AIF quality check and were excluded. Finally, a total of 1843 cases (mean age of 74 ± 14 years, 879 females) were included in the final analysis. Within this group, a subset of 868 cases had echocardiography reports available which included measurement of the ejection fraction (mean EF 54 ± 7%).

**Fig. 2:**
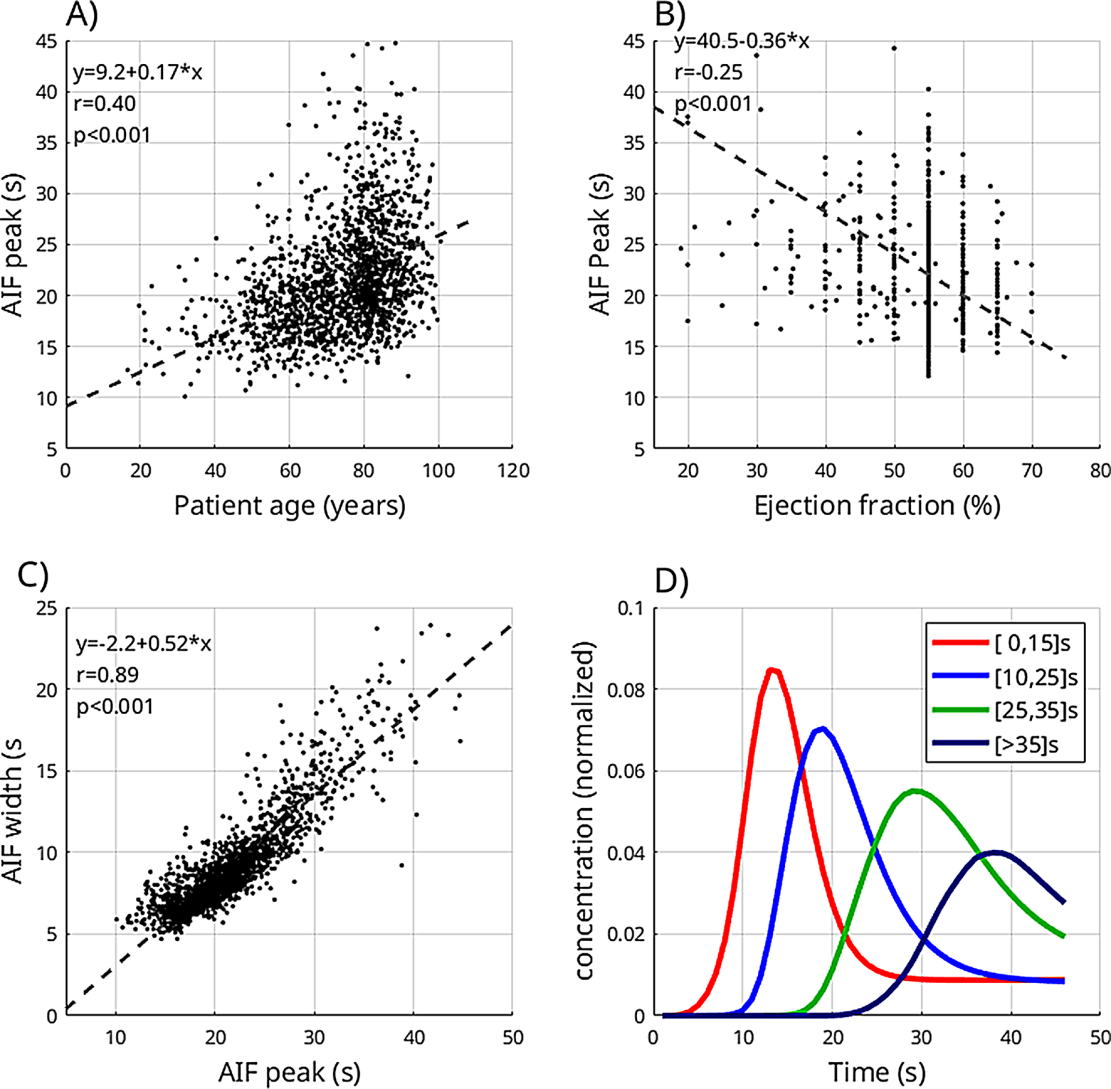
CTP bolus characteristics. Patient individual factors (age and ejection fraction) and arterial input function (AIF) width were significantly associated with AIF peak position (**A-B**). AIF peak position and the peak width strongly correlated (**C**), indicating a coupled effect of bolus delay and dispersion. This is supported by averaged time concentration curves of all patients within a specific bolus arrival category (**D**). Here, curves with later bolus arrival denote increased dispersion

Exceeding the injector’s pressure limit can reduce the flow rate and increase the flow duration. Indeed, in 512 cases, the nominal flow durations of 14 s for CTA and 6.6 s for CTP were exceeded. However, the increase was typically minor (mean ± SD = 1 ± 0.8 s), so we expect only a minor impact on the bolus in our study, contributing to general noise.

### CTP bolus characteristics

CTP bolus characteristics are summarized in Fig. 2. We noted significant correlations between CTP AIF peak position and peak width (Pearsons’s *r* = 0.89, *p* < 0.001). CTP AIF peak time was weakly associated with patient age (Pearsons’s *r* = 0.40, *p* < 0.001) and ejection fraction (Pearsons’s *r*=-0.25, *p* < 0.001).

The multivariable analysis revealed that age, ejection fraction, and the CTA scan delay were significantly associated with the CTP AIF peak position (*p* < 0.002), whereas no significant association was present for sex or weight (*p* > 0.41).

**Fig. 3:**
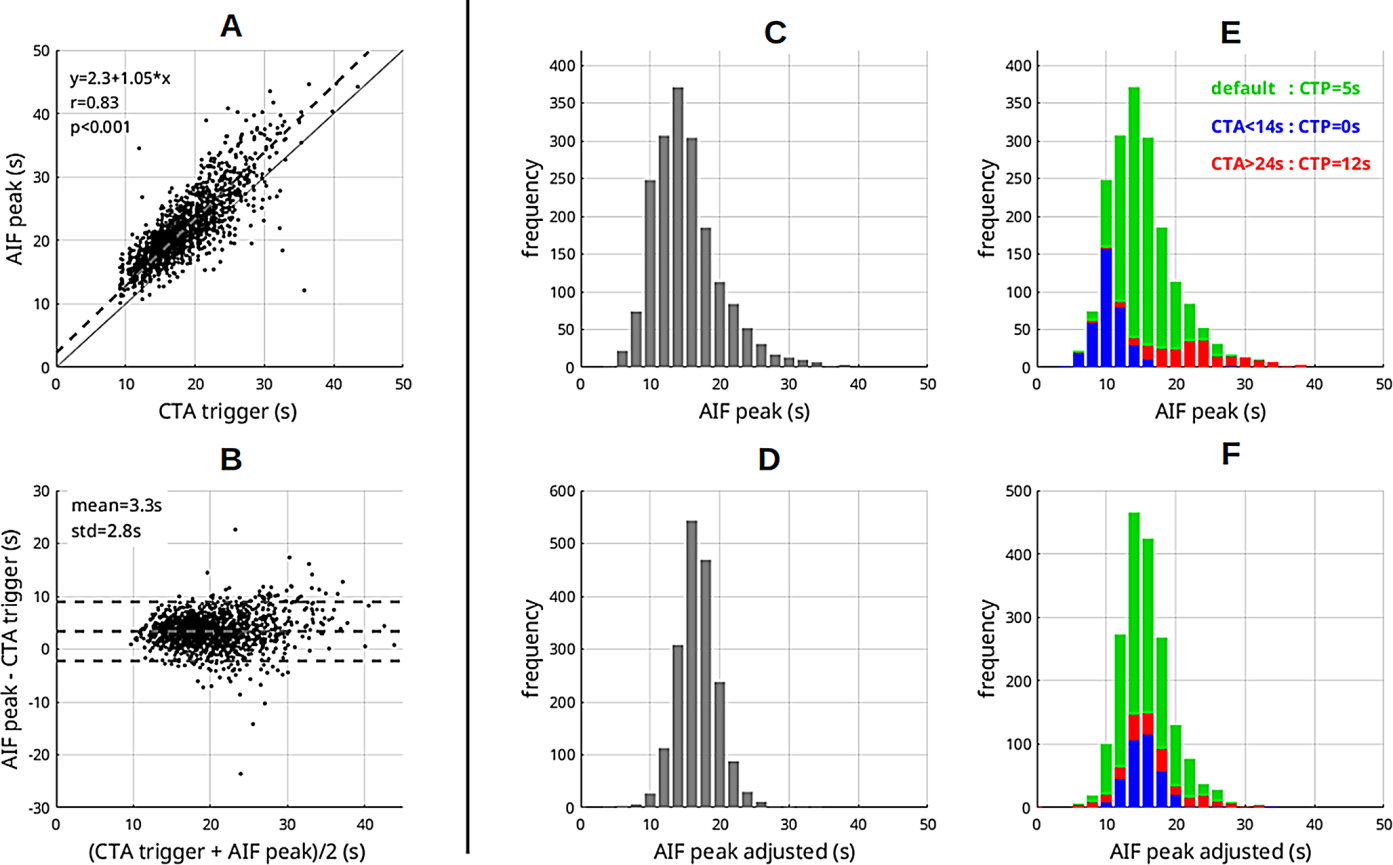
Optimization of CTP timing. Correlation plot (**A**) and Bland-Altman plot (**B**) show the dependency between CTA trigger time and CTP AIF peak position. This relationship was used to simulate an optimized CTP timing using the CTA trigger time based on automated and manual adjustment strategies. The variability of peak time distribution for the original, fixed delay of 5s (**C**) could be significantly reduced with the automated adjustment (**D**), which shows a narrower distribution. Panel **E** illustrates the logic of the rule-based adjustment: Cases with normal CTA trigger (green) would be started with the default CTP delay of 5s, whereas cases with early CTA trigger (blue) would be started with zero delay, and cases with late CTA trigger (red) would be started with an extended delay of 12s. Also for this rule-based approach, a reduction in the variability of peak positions could be achieved (**F**)

**Fig. 4:**
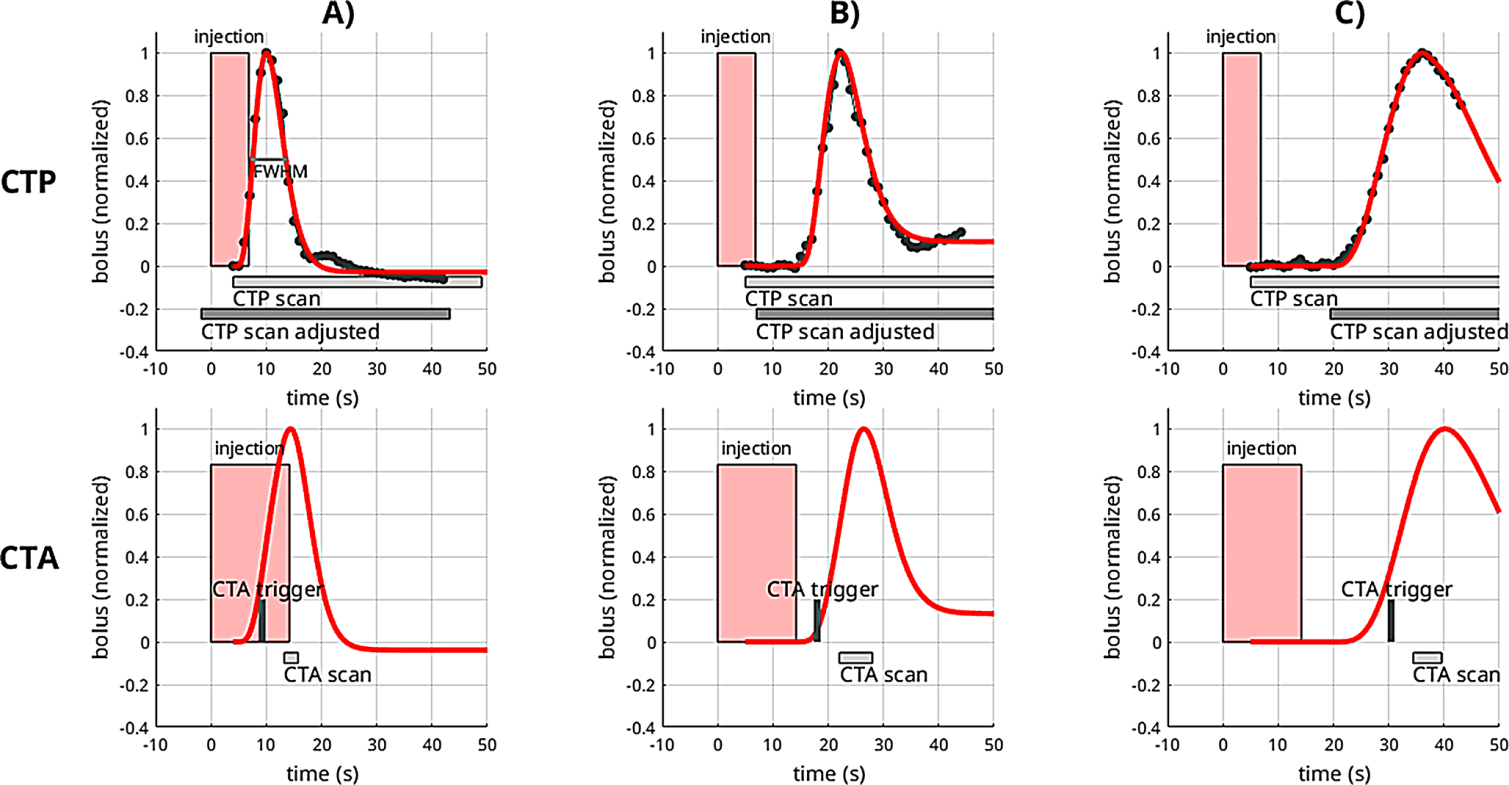
Illustration of optimized CTP bolus timing and simulated CTA arterial attenuation curves. CTP scans are shown in the top and CTA in the bottom row in three different subjects with early (**A**), accurate (**B**), and late bolus arrival (**C**). Red boxes represent bolus injections. Black dotted lines and red curves show the measured and fitted AIF, respectively. For the CTA scan, AIF curves were simulated using a convolution to account for different injection schemes in CTP and CTA. Gray bars show the actual acquisition window. The dark gray bars in CTP depict the optimized acquisition window using the patient-individual CTA trigger time instead of a fixed delay which would result in an optimized CTP acquisition

**Fig. 5:**
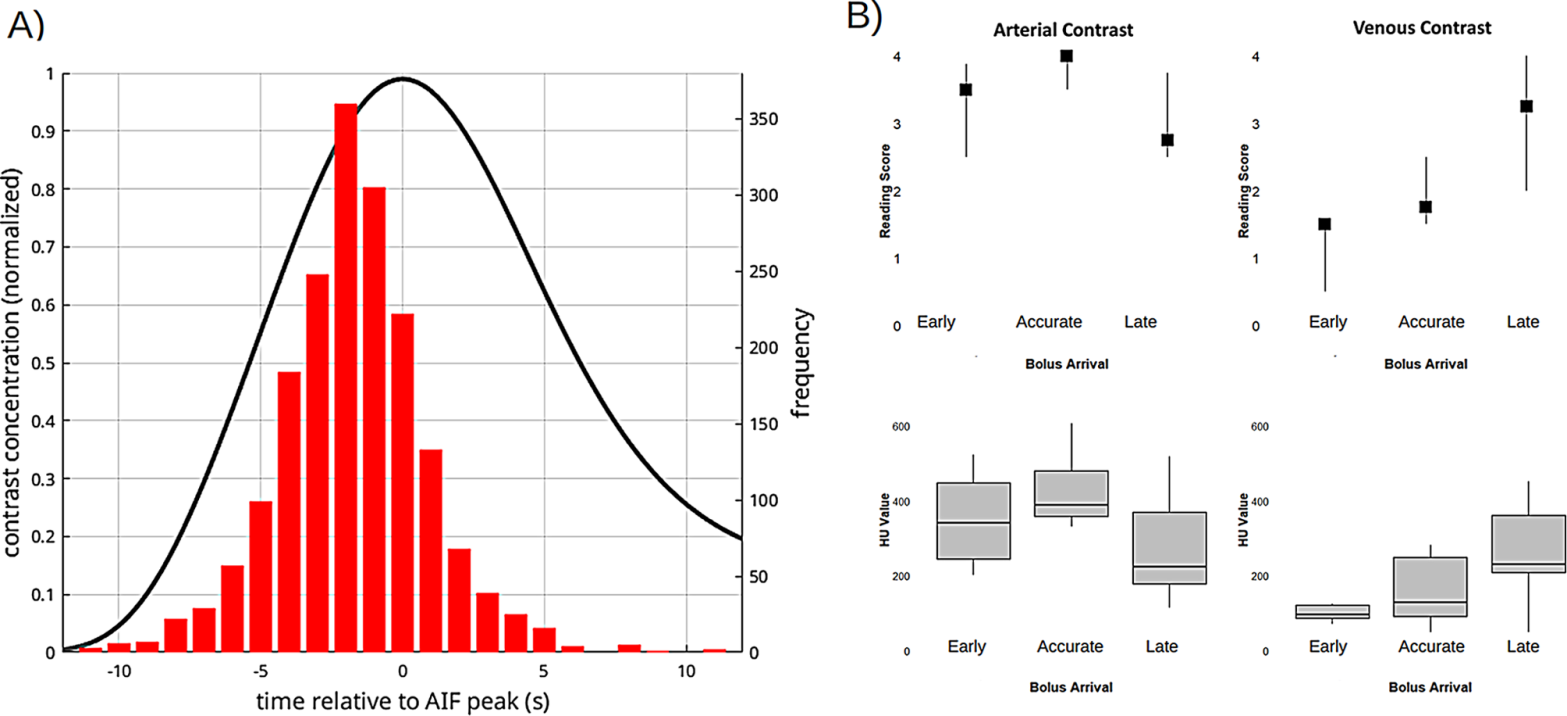
(**A**) Distribution of CTA timing. The black curve represents the mean of all arterial curves centered around their maximum position. The red histogram depicts the distribution of the offsets of the actual CTA scan time with respect to the arterial peak. A high negative offset indicates that the CTA was triggered too early, a zero or slightly negative offset corresponds to accurate triggering and a positive offset indicates too late triggering. (**B**) Results from visual and quantitative assessment of 10 early, 10 accurate, and 10 late scans: Both, the visually scored and measured attenuation in the ICA showed a pattern similar to the arterial curve, where the highest score and contrast is reached at the peak with “accurate timing”. For the venous scores and attenuation measurements, the contrast attenuation was increasingly higher with later triggering

**Fig. 6:**
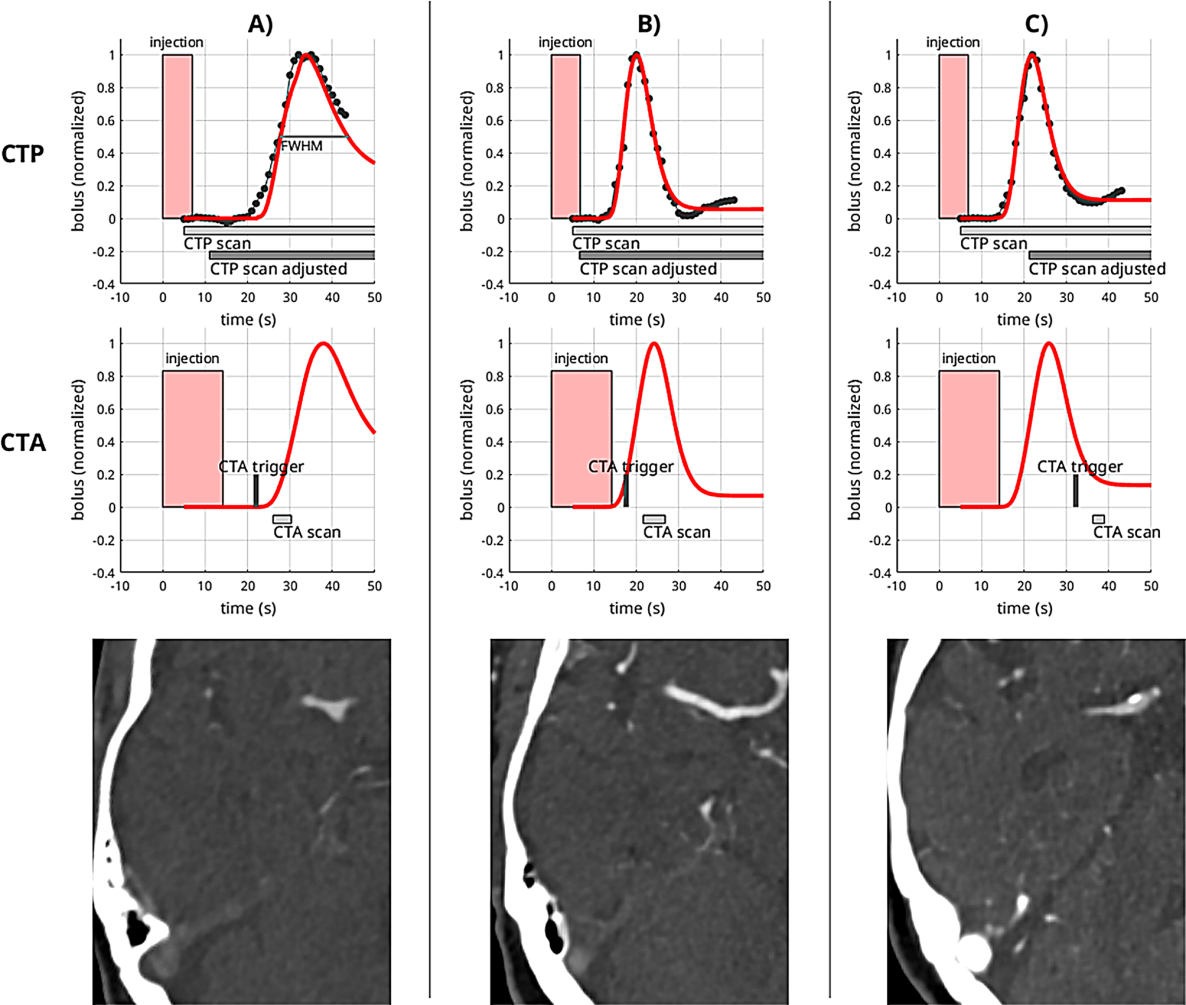
Exemplary cases of different CTA timing. For curve legend see Fig. 4. A comparison of the CTP curves and CTA timing reveals that CTA was triggered too early (**A**), just right (**B**), and too late (**C**) with respect to the actual arterial bolus. This finding is supported by visual inspection of the corresponding CTA images: ideal contrast is achieved with optimal timing, while venous contrast becomes apparent with delayed triggering, necessitating cautious interpretation of CTA images in such scenarios. Moreover, in such instances, adopting the CTA delay for adjusted CTP timing could exacerbate rather than ameliorate timing discrepancies

### Optimized CTP timing

A strong association between CTP AIF peak position, and the CTA trigger time was found (Pearsons’s *r* = 0.83, *p* < 0.001), Fig. 3A and B. Using the information on this relationship to optimize the CTP timing resulted in narrower histograms as given in Fig. 3 (C-E). The standard deviation decreased from 4.9s for the fixed delay to 2.7s for the automated adjustment, and 3.4s for the rule-based adjustment. In the analysis of outliers exhibiting very early or late bolus arrival, we found that the AIF peak fell outside the acceptable range of 15 to 30 s in 11.5% of cases with the original fixed scan delay. For the automated correction method, this figure decreased substantially to 1.7%, while the rule-based approach resulted in 4.3% of cases outside the optimal range.

Figure 4 provides a detailed illustration of the CTP timing and optimized results for cases with very early, accurate, and late CTP bolus arrival.

### Optimized CTA timing

The distribution of the differences between the actual CTA scan, and the simulated CTA arterial curve is shown in Fig. 5A together with the mean of all CTA AIF curves centered around the maximum position. The results indicate that the CTA was acquired in most cases at the rising flank of the bolus curve, shortly before the peak. However, the distribution showed substantial variability and the frequent occurrence of suboptimal (too early or too late) timing, i.e. a substantial offset.

A negative offset indicates that the CTA was triggered too early, zero offset represents accurate timing and a positive offset indicates too late triggering.

The results from the visual and quantitative assessment of the 30 cases with early, accurate, and late timing (see Fig. 5B) supported this assumption: Both raters consistently attributed the highest arterial score to the accurately timed scans, and the highest venous score to the lately triggered scans. Accordingly, the attenuation in the ICA was highest for the scans with presumed accurate timing. In contrast to this, the attenuation in the sigmoid sinus was highest for the late-trigged scans.

## Discussion

In this study, we investigated the injection- and scan timing of CTA and CTP scans in a large clinical dataset to gain information on the impact on the bolus architecture and investigate the relevance of imaging contrast. This work yielded several noteworthy conclusions.

First, we found substantial variability in bolus arrival times and coupled delay and dispersion in the concentration curves in both CTA and CTP. This variability was significantly associated with cardiac ejection fraction, thus is primarily influenced by cardiac output. Reduced cardiac output can lead to slower mixing of the contrast agent within the cardiovascular system, resulting in delayed bolus arrival and increased dispersion, as indicated in Fig. 6. The correlation between the peak position and age also aligns logically, given the well-established relationship between age and cardiac output [22].

It is known that the variability in contrast arrival is a pitfall in CTP imaging. The idea to use patient-specific trigger times for CTP acquisition from CTA bolus tracking instead of a fixed delay is not new and has been demonstrated on a rather small patient cohort with rule-based triggering [10]. In our retrospective study using a large dataset with a great variety of bolus shapes, we tested on generated models how an optimized CTP timing would impact the CTP bolus arrival. We found that the optimization substantially reduced the proportion of improperly timed CTP scans. The automated method was superior to the rule-based one. These findings advocate for the integration of automatic forwarding of CTA trigger times to CTP protocol by vendors.

Due to the aforementioned coupling of delay and dispersion, not only should the CTP delay be adjusted, but it seems imperative to also extend the acquisition window for delayed arrivals to ensure that the bolus is fully captured, which aligns with recommendation made by previous studies [10, 16]. A patient-individual adaptation of the scan duration might therefore contribute to a reduction in the number of uninterpretable CTP scans and optimized radiation dose.

In addition to utilizing CTA triggers for optimizing CTP scans, we gained some valuable insights in the reverse direction. Comparing CTA scan times with simulated arterial peaks from CTP AIF revealed that, on average, scans were optimally timed shortly before the arterial peak. However, we also identified instances of suboptimal CTA timing. We attribute this to factors such are misplacement of ROI for triggering, patient movement, image noise, and the known limitations of a single HU threshold for triggering. These findings underscore the importance of recognizing that CTA timing may not always be optimal even when bolus tracking is applied. Moreover, this underlines the necessity of careful CTA assessment, especially the collateral assessment, either performed manually or with specialized software.

In this context, it’s important to note that suboptimal CTA triggering can adversely affect CTP outcomes when implementing an adjustment method such as the one proposed in this study. In these cases, the CTP may not be improved but worsened, as illustrated in Fig. 6C.

Although suggestions to improve CTA such as implementing multiphasic plateau-based injection schemes [23] exist, their complexity has hampered their widespread adoption in clinical practice. Improved monitoring of bolus tracking curves, such as the curve fitting to contrast rise instead of single threshold methods [23], or the automatic selection of optimal ROI in the aortic arch, could offer other promising approaches for refinement. Furthermore, multi-phase CTA (mCTA) or dynamic CTA have increasingly become established in acute stroke imaging protocols as they combine benefits of both CTA and CTP [24]. Our findings hold relevance in this context as well, especially since precise timing is even more crucial when fewer time points are acquired within a shorter temporal acquisition window in mCTA.

Though our results might impact diagnostic routine, limitations of our study require further investigations. First, we used a relatively short acquisition window of 45 s for CTP to mitigate radiation dose. In general, shorter windows increase the likelihood of known issues such as bolus truncation. Therefore, some studies advocate for longer windows of 60 s to eliminate this error [15]. On the other hand, longer windows result in significantly higher radiation doses and thus a compromise has to be found. Our findings suggest that adaptive triggering using the CTA delay could provide a solution on an individual patient-level. Second, we excluded cases with bolus peaks occurring after the acquisition window (i.e. severe bolus truncations) because no reasonable curve fit could be performed in such cases. Consequently, the actual number of late contrast arrivals is underestimated in our study.

It should be further noted that the proposed correction method can only be applied when CTP follows CTA. This order is often preferred in a timely workflow, as CTA images can be assessed during CTP acquisition [25]. Potential contrast bolus interferences can be avoided with a sufficient delay between CTA and CTP and/or correction algorithms [18]. Fully automated CTP triggering could further help by automatically ensuring the proper delay.

We did not explicitly explore our dataset for confounding factors like severe carotid stenosis and others. It is reasonable to assume that such cases were present within our dataset. However, the implications of these factors remain unclear and warrant further exploration. Moreover, the injection site may contribute to different bolus arrival geometry.

Though our study is retrospective in nature, we consider this a strength, since we could investigate the optimization in detail based on a rich clinical dataset that was already available, whereas a prospective study would necessitate a substantial dataset to capture both early and late arrivals comprehensively.

In summary, our exploration of CTP and CTA timing based on a comprehensive dataset from clinical practice, reveals significant variability in bolus arrivals, largely attributed to patient-specific cardiac output. Our findings suggest that CTP timing can be improved by leveraging timing data from preceding CTA scans.
